# Protocatechuic aldehyde ameliorates high glucose-induced podocyte injury by attenuating inflammation, oxidative stress, and apoptosis *via* suppression of endoplasmic reticulum stress through the GSK3β/Nrf2 pathway

**DOI:** 10.3389/fcell.2025.1693955

**Published:** 2025-11-11

**Authors:** Yishu Wang, Haifeng Wang, Yang Li

**Affiliations:** 1 Comprehensive Internal Medicine Department, Beijing Xiaotangshan Hospital, Beijing, China; 2 Department of Nephrology, China-Japan Friendship Hospital, Beijing, China

**Keywords:** podocytes, endoplasmic reticulum stress, GSK3β/Nrf2 signaling pathway, apoptosis, oxidative stress, inflammation, protocatechuic aldehyde

## Abstract

**Background:**

The core pathological feature of Diabetic kidney disease is glomerular podocyte injury. A hyperglycemic milieu induces podocyte injury through the synergistic actions of multiple pathways, including oxidative stress, inflammation, and apoptosis. Protocatechuic Aldehyde (PCA), a naturally occurring phenolic acid compound, exhibits significant antioxidant activity. However, the protective effects and underlying mechanisms of PCA on podocyte function under high-glucose conditions remain incompletely elucidated.

**Objective:**

To investigate the effects and mechanism of PCA on high glucose-induced podocyte inflammation, oxidative stress, and apoptotic injury.

**Methods:**

A podocyte injury model was established by treating mouse podocytes (MPC5) with high-glucose medium. Podocytes were concurrently treated with varying concentrations of Protocatechuic Aldehyde. To explore the mechanism, cells in different treatment groups were exposed to the GSK3β inhibitor TDZD-8 and the endoplasmic reticulum stress inducer Tunicamycin (TM). The levels of inflammatory cytokines and oxidative stress markers were measured using relevant assay kits. The expression of proteins associated with inflammation, oxidative stress, apoptosis, the GSK3β/Nrf2 signaling pathway, and endoplasmic reticulum stress was detected by Western blot. Apoptosis rate of podocytes was assessed using flow cytometry.

**Results:**

High glucose significantly reduced MPC5 cell viability and increased lactate dehydrogenase release; these effects were significantly reversed by PCA treatment. PCA significantly reduced the secretion of inflammatory cytokines (TNF-α, IL-1β, IL-6), restored the activities of SOD and GSH-Px, decreased MDA content, and downregulated the expression of Cox-2, iNOS, Nox2, and Nox4 proteins, thereby suppressing HG-induced podocyte inflammation and oxidative stress. Furthermore, PCA upregulated Bcl-2 expression while downregulating Bax and cleaved-caspase 3 expression, effectively inhibiting HG-induced podocyte apoptosis. Mechanistically, PCA upregulated the expression of p-GSK3β and Nrf2 proteins, activating the GSK3β/Nrf2 signaling pathway. This activation was associated with downregulation of ER stress markers (CHOP, GRP78, p-PERK), indicating suppression of podocyte ER stress. Notably, the protective effects of PCA were abrogated by co-treatment with the GSK3β inhibitor TDZD-8 or the ER stress inducer TM.

**Conclusion:**

PCA attenuates high glucose-induced podocyte injury, characterized by inflammation, oxidative stress, and apoptosis, suggesting that this protection involves inhibition of ER stress *via* activation of the GSK3β/Nrf2 signaling pathway.

## Graphical Abstract

**Figure:**
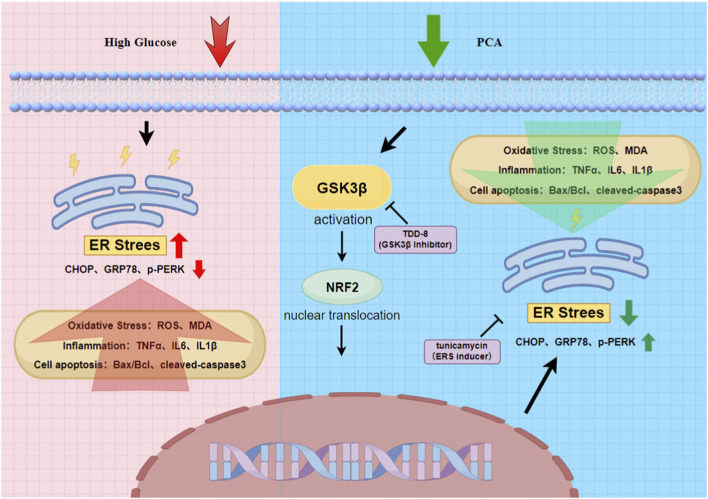
PCA can inhibit ERS through the GSK3β/Nrf2 signaling pathway, thereby ameliorating high glucose-induced inflammation, oxidative stress, and apoptotic damage in podocytes.

## Introduction

Diabetic kidney disease (DKD) is one of the most severe microvascular complications of diabetes mellitus and a leading cause of ESKD. Within the pathogenesis of DKD, glomerular podocyte injury constitutes a pivotal event (Reidy et al., 2014). A hyperglycemic milieu induces podocyte damage through multiple pathways: On one hand, it triggers excessive reactive oxygen species (ROS) production, disrupting redox homeostasis and inducing oxidative stress; on the other hand, it activates inflammatory cascades and pro-apoptotic signaling pathways, ultimately leading to the structural and functional demise of podocytes (Susztak et al., 2006).

Endoplasmic reticulum stress (ERS) is a key mechanism in hyperglycemia-induced podocyte injury. Sustained hyperglycemia causes sustained overactivation of the unfolded protein response, particularly the PERK-eIF2α and IRE1-XBP1 pathways. This exacerbates oxidative stress and elicits apoptosis *via* CHOP-dependent pathways (Cybulsky, 2010). Studies demonstrate that hyperactivation of GSK3β (phosphorylation at Tyr216) suppresses the nuclear translocation of Nrf2, thereby impairing the expression of antioxidant genes (like HO-1, NQO1) (Lu et al., 2019). Clinical evidence reveals significantly elevated GSK3β activity alongside suppressed Nrf2 activity in renal biopsy samples from DKD patients, highlighting the therapeutic potential of targeting this pathway (Mohs et al., 2021).

Protocatechuic Aldehyde (PCA), a naturally occurring phenolic acid compound found in medicinal plants such as Salvia miltiorrhiza (Danshen), has been primarily investigated for its hepatoprotective effects (Song et al., 2025). Previous research confirmed PCA’s ability to mitigate cyclophosphamide-induced hepatic oxidative stress and apoptosis *via* Nrf2 pathway activation. However, its role in protecting podocytes in DKD remains undefined.

Existing research indicates that GSK3β inhibitors significantly reduce proteinuria in adriamycin nephropathy models, exerting podocyte protection in an Nrf2 activation-dependent manner. Furthermore, miR-378c alleviates high glucose-induced podocyte apoptosis by suppressing GSK3β and consequently upregulating Nrf2, suggesting the druggability of the GSK3β-Nrf2 axis. Concurrently, PCA has demonstrated Nrf2-activating and anti-ERS effects in liver injury models (Feng et al., 2024). Based on these findings, we hypothesized that PCA protects against high glucose-induced podocyte injury by inhibiting GSK3β, thereby activating Nrf2 and suppressing endoplasmic reticulum stress. This study aims to validate the molecular mechanism by which PCA regulates ERS *via* the GSK3β/Nrf2 pathway, offering novel therapeutic strategies for diabetic kidney disease.

## Materials and methods

### Cells

The mouse podocyte cell line (MPC5) was provided by the Shanghai Institute of Biochemistry and Cell Biology, Chinese Academy of Sciences (Shanghai, China).

## Reagents

Protocatechuic Aldehyde (PCA) was purchased from Baoji Herbest Bio-Tech Co., Ltd (Baoji, Shaanxi, China).The GSK3β inhibitor TDZD-8 (HY-11012) and the endoplasmic reticulum stress inducer Tunicamycin (TM, HY-A0098) were both purchased from MedChemExpress (MCE) China.Dulbecco’s Modified Eagle Medium (DMEM) and fetal bovine serum (FBS) were purchased from Thermo Fisher Scientific.The MTT Cell Proliferation and Cytotoxicity Assay Kit (C0009) and the Lactate Dehydrogenase (LDH) Cytotoxicity Assay Kit (C0017) were purchased from Beyotime Biotechnology (Shanghai, China).The Superoxide Dismutase (SOD) Activity Assay Kit (BC0175), Glutathione Peroxidase (GSH-Px/GPX) Activity Assay Kit (BC1195), and Malondialdehyde (MDA) Content Assay Kit (BC0025) were purchased from Solarbio Life Sciences (Beijing, China).Mouse-specific Enzyme-Linked Immunosorbent Assay (ELISA) Kits for Tumor Necrosis Factor-alpha (TNF-α), Interleukin-1 beta (IL-1β), and Interleukin-6 (IL-6) were purchased from Yuanye Bio-Technology Co., Ltd. (Shanghai, China), Detailed information regarding primary antibodies, incubation conditions, and secondary antibodies is provided in Supplementary Table 1.

## Cell culture and treatment

Mouse podocyte clone 5 (MPC5) cells were cultured in high-glucose Dulbecco’s Modified Eagle Medium [HG-DMEM; containing 10% fetal bovine serum (FBS), 1% penicillin-streptomycin, and 10 U/mL interferon-gamma (IFN-γ)] at 33 °C under a humidified atmosphere of 5% CO_2_. To induce podocyte differentiation, the cells were shifted to an incubator at 37 °C and cultured in medium devoid of IFN-γ for 14 days. Mature, differentiated podocytes were then used for subsequent experiments. For HG treatment, differentiated MPC5 cells were exposed to DMEM containing 30 mM D-glucose for 24 h. The control group was maintained in normal glucose DMEM containing 5.5 mM D-glucose. For MAN treatment, referred to the D-Mannose control group, with its concentration matching the high-glucose group. For PCA treatment, cells were concomitantly treated with PCA (1.25, 2.5, or 5.0 μM) for 24 h during HG exposure. For mechanistic studies involving TDZD-8 (5 μM) or tunicamycin (TM, 2 μg/mL), cells were pretreated for 2 h prior to HG and PCA exposure.

### MTT assay for cell viability

MPC5 cells were seeded into 96-well plates at a density of 1 × 10^4^ cells per well and incubated for 24 h. Following treatment according to the experimental group designations, 10 μL of MTT solution (5 mg/mL) was added to each well, and the cells were incubated for an additional 4 h at 37 °C. Subsequently, 150 μL of dimethyl sulfoxide (DMSO) was added per well to dissolve the formazan crystals. The absorbance was measured at a wavelength of 490 nm using a microplate reader.

### Assessment of cytotoxicity using lactate dehydrogenase (LDH) cytotoxicity assay kit

MPC5 cells were seeded into 96-well plates and allowed to grow to 80%–90% confluency. Cells were treated with various drugs according to the experimental groups, with appropriate controls included. Following treatment, the culture plates were centrifuged at 400 × g for 5 min. The supernatant was aspirated, and 150 μL of LDH release reagent (diluted in PBS) was added to each well. Plates were then incubated for 1 h under standard culture conditions. Subsequently, plates were centrifuged again, and 120 μL of supernatant from each well was carefully transferred to corresponding wells of a new 96-well plate. The absorbance was measured at 490 nm. LDH activity in each group was calculated based on a concurrently generated LDH standard curve.

### Measurement of inflammatory cytokine levels using ELISA kits

The secretion levels of inflammatory cytokines (TNF-α, IL-1β, and IL-6) in the cell culture supernatants of different groups were quantified using specific Enzyme-Linked Immunosorbent Assay (ELISA) kits strictly following the manufacturer’s instructions. The optical density (OD) was measured at a wavelength of 450 nm using a microplate reader. Cytokine concentrations in the culture medium were calculated using the respective standard curves and the absorbance values obtained for each well.

### Evaluation of oxidative stress levels using biochemical assay kits

The activities of superoxide dismutase (SOD) and glutathione peroxidase (GSH-Px), as well as the content of malondialdehyde (MDA), in cell lysates were measured using specific biochemical assay kits according to the manufacturers’ protocols. Absorbance readings were taken using a microplate reader at the following wavelengths: 560 nm for SOD, 412 nm for GSH-Px, and 532 nm/600 nm (dual-wavelength measurement) for MDA.

### Analysis of protein expression by western blotting

Cells were harvested and lysed on ice for 30 min using RIPA lysis buffer supplemented with phenylmethylsulfonyl fluoride (PMSF) at a ratio of RIPA:PMSF = 100:1. Lysates were centrifuged at 4 °C, and the supernatant was collected. Protein concentration was determined using a Bicinchoninic Acid (BCA) Protein Assay Kit. Protein samples were mixed with loading buffer and denatured. Proteins were separated by electrophoresis on 10% SDS-polyacrylamide gels (SDS-PAGE) and then transferred onto polyvinylidene difluoride (PVDF) membranes (Millipore, United States). Membranes were blocked with 5% bovine serum albumin (BSA) or 5% non-fat dry milk in Tris-buffered saline with Tween 20 (TBST) for 2 h at room temperature. Subsequently, membranes were incubated with primary antibodies overnight at 4 °C. The following day, membranes were washed and incubated with appropriate species-specific horseradish peroxidase (HRP)-conjugated secondary antibodies for 1 h at room temperature. After thorough washing with TBST, protein bands were visualized using enhanced chemiluminescence (ECL) substrate and detected using a chemiluminescence imaging system. Band density was quantified using ImageJ software. Nuclear and cytoplasmic proteins were separated using a commercial extraction kit (P0028). The isolated nuclear fractions were used to assess NRF2 levels, as nuclear localization of NRF2 represents the transcriptionally active form of the protein.

### Quantification of apoptosis by flow cytometry

Following trypsinization and centrifugation, harvested cells were washed with ice-cold phosphate-buffered saline (PBS) and resuspended to form a single-cell suspension. Each sample was stained with Annexin V-Fluorescein Isothiocyanate (Annexin V-FITC; 5 μL) and Propidium Iodide (PI; 10 μL), followed by incubation at room temperature for 15 min in the dark. The stained cell suspension was transferred to flow cytometry tubes and analyzed using a flow cytometer within 1 h.

### Statistical analysis

Data are expressed as mean ± SD from three biologically independent experiments. Statistical analysis was conducted using GraphPad Prism version 9.0, with Student’s t-test and one-way ANOVA employed for group comparisons. A p-value <0.05 was considered statistically significant.

## Results

### PCA attenuates high glucose-induced podocyte injury

**FIGURE 1:**
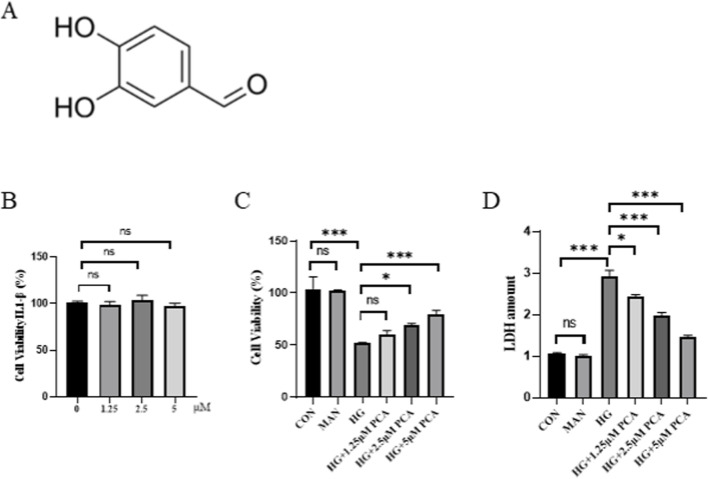
PCA attenuates high glucose-induced podocyte injury. **(A)** Chemical Structure of PCA. **(B)** Effects of different concentrations of PCA on cell viability **(C)** Effects of HG induction and subsequent PCA treatment on cell viability. **(D)** Effects of HG induction and subsequent PCA treatment on cell cytotoxicity. Data are presented as the mean ± SD, n = 3. * *P* < 0.05, ** *P* < 0.01, *** *P* < 0.001.

Protocatechuic Aldehyde (PCA), also known as 3,4-dihydroxybenzaldehyde, is a water-soluble phenolic acid compound naturally present in the medicinal plant *Salvia miltiorrhiza*. Its chemical structure is shown in Figure 1A. Initially, the effect of different concentrations of PCA on MPC5 cell viability was assessed using the MTT assay to determine an appropriate non-toxic concentration range. Results demonstrated that treatment with PCA at concentrations of 1.25, 2.5, and 5.0 μM did not significantly affect cell viability (Figure 1B). Subsequently, a podocyte injury model was established by stimulating cells with high glucose. Compared with the control group, HG stimulation significantly reduced cell viability and significantly increased LDH release (Figure 1C), indicating successful induction of podocyte injury. Treatment with PCA significantly increased cell viability and reduced LDH levels compared to the HG group (Figure 1D). These findings demonstrate that PCA effectively attenuates high glucose-induced podocyte injury.

### PCA attenuates high glucose-induced podocyte inflammation and oxidative stress

**FIGURE 2:**
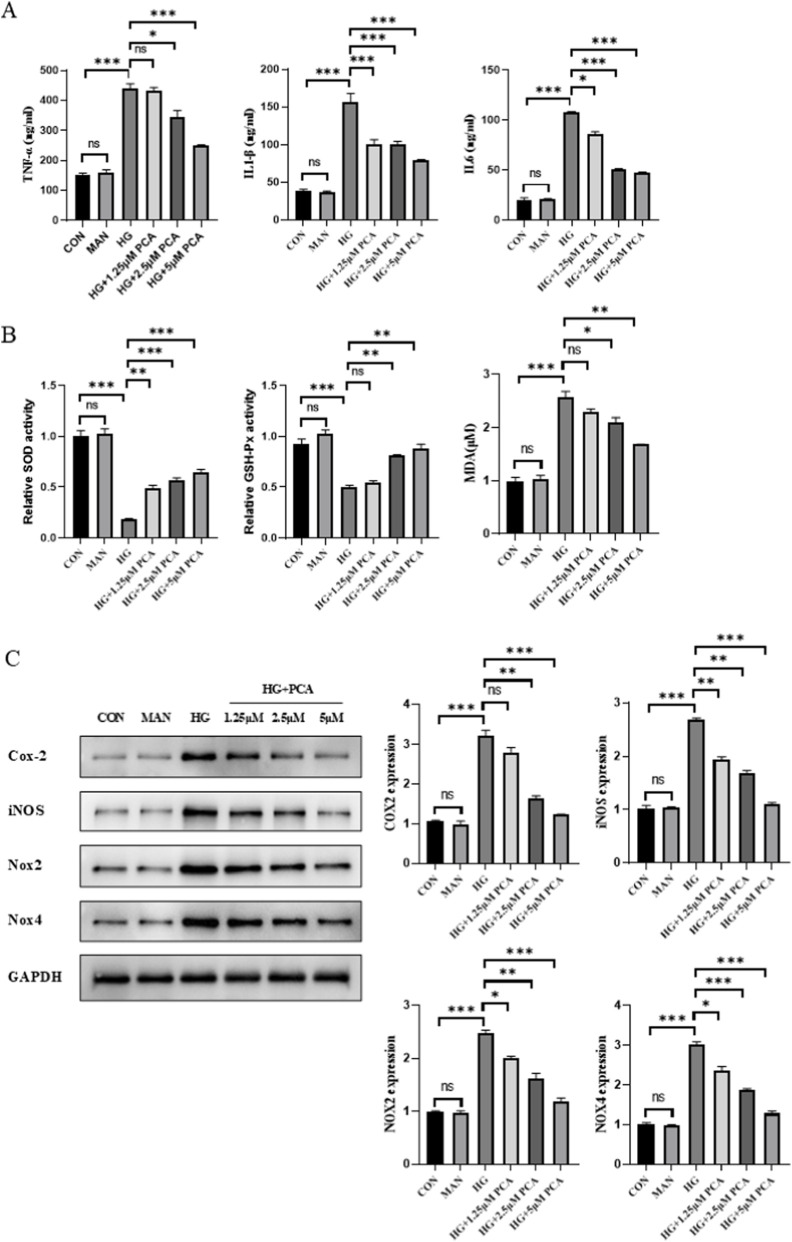
PCA attenuates high glucose-induced podocyte inflammation and oxidative stress. **(A)** Levels of inflammatory cytokines (TNF-α, IL-1β, and IL-6) in cells were measured using ELISA kits. **(B)** Activities of SOD and GSH-Px, as well as MDA content, were determined using biochemical assay kits. **(C)** Expression levels of proteins associated with inflammation and oxidative stress (Cox-2, iNOS, Nox2, and Nox4) were detected by Western blot. Data are presented as the mean ± SD, n = 3. * *P* < 0.05, ** *P* < 0.01, *** *P* < 0.001.

To investigate the effects of PCA on inflammation and oxidative stress in podocytes under HG conditions, the levels of inflammatory cytokines were first measured using ELISA. Results demonstrated that compared to the control group, HG stimulation significantly upregulated the levels of inflammatory cytokines TNF-α, IL-1β, and IL-6 in MPC5 cells. Treatment with PCA significantly inhibited the secretion of these inflammatory cytokines (Figure 2A). Concurrently, cellular oxidative stress levels were assessed. Experimental data revealed that HG treatment significantly reduced the activities of superoxide dismutase (SOD) and glutathione peroxidase (GSH-Px), while elevating malondialdehyde (MDA) levels compared to the control group. PCA treatment effectively restored SOD and GSH-Px activities and reduced MDA content (Figure 2B). Furthermore, the expression of proteins associated with inflammation and oxidative stress was analyzed by Western blotting. Results showed that the expression of cyclooxygenase-2 (Cox-2), inducible nitric oxide synthase (iNOS), NADPH oxidase 2 (Nox2), and NADPH oxidase 4 (Nox4) proteins was significantly increased in the HG group compared to controls. PCA treatment significantly reduced the expression of Cox-2, iNOS, Nox2, and Nox4 proteins (Figure 2C).

Collectively, these results demonstrate that PCA effectively attenuates high glucose-induced inflammation and oxidative stress in podocytes.

### PCA inhibits high glucose-induced podocyte apoptosis

**FIGURE 3:**
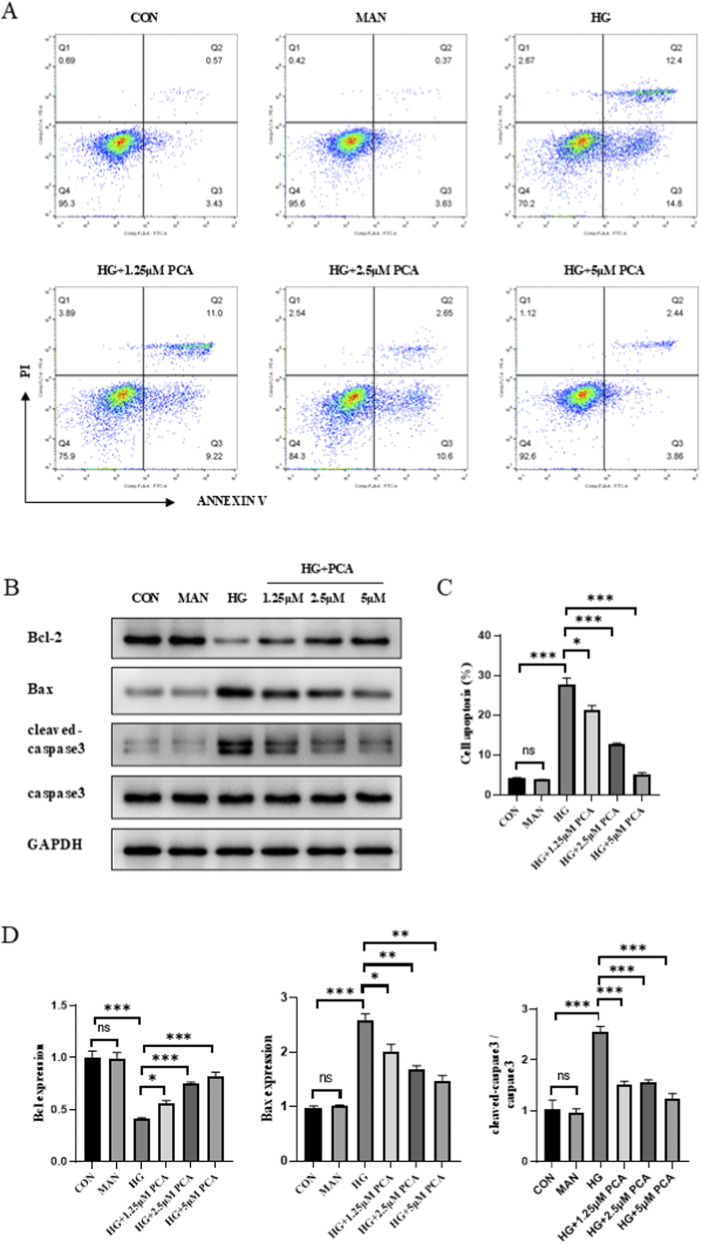
PCA inhibits high glucose-induced podocyte apoptosis. **(A, C)** Apoptosis was detected by flow cytometry. **(B, D)** The expression of apoptosis-related proteins Bcl2, Bax, and cleaved caspase 3 was measured by Western blot. Data are presented as the mean ± SD, n = 3. *P < 0.05, **P < 0.01, ***P < 0.001.

The effect of PCA on HG-induced podocyte apoptosis was assessed using flow cytometry. Results demonstrated that compared to the control group, HG treatment significantly increased the podocyte apoptosis rate. PCA intervention significantly reduced HG-induced apoptosis in a dose-dependent manner (Figure 3A). Furthermore, Western blot analysis revealed that compared to controls, HG stimulation significantly downregulated the expression of the anti-apoptotic protein Bcl-2 while significantly upregulating the expression of the pro-apoptotic proteins Bax and cleaved-caspase 3 in podocytes. However, compared to the HG group, PCA treatment significantly increased Bcl-2 protein expression and significantly decreased the expression of both Bax and cleaved-caspase 3 proteins (Figure 3B). Collectively, these findings indicate that PCA inhibits high glucose-induced podocyte apoptosis by modulating the expression of key apoptosis-related proteins, including Bcl-2, Bax, and cleaved-caspase 3.

### PCA suppresses endoplasmic reticulum stress by activating the gsk3β/nrf2 signaling pathway

**FIGURE 4:**
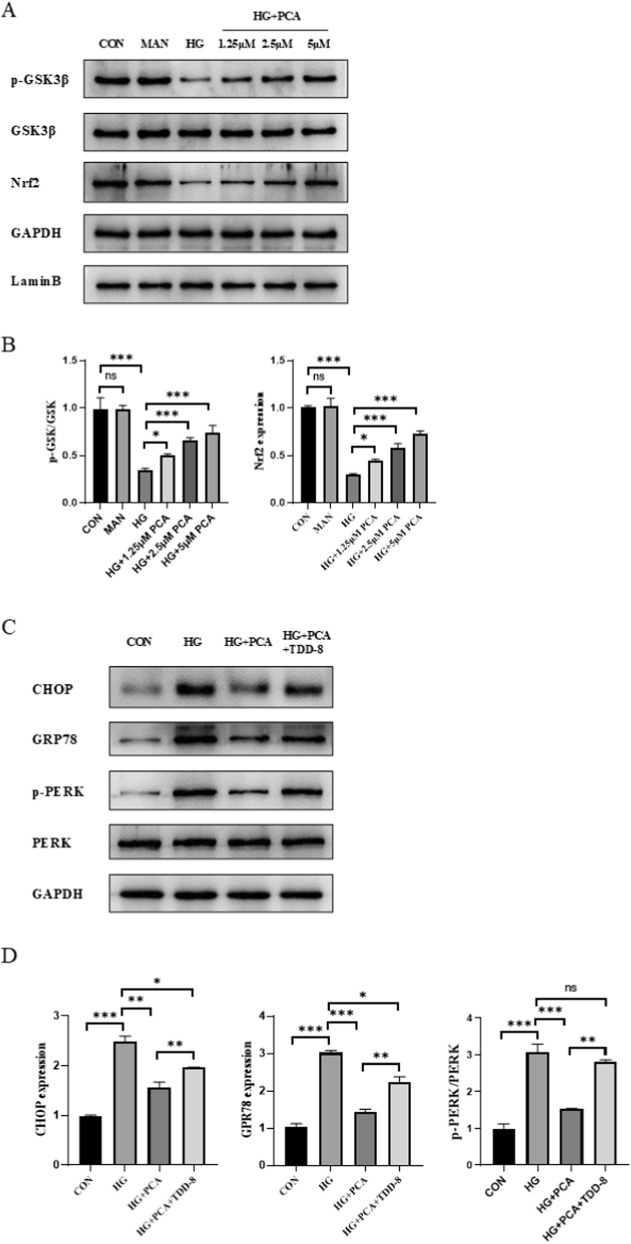
PCA suppresses endoplasmic reticulum stress by activating the gsk3β/nrf2 signaling pathway. **(A, B)** The protein expression levels associated with the GSK3β/Nrf2 signaling pathway were analyzed by Western blot. **(C, D)** The expression of proteins related to endoplasmic reticulum stress was evaluated by Western blot. Data are presented as the mean ± SD, n = 3. * *P* < 0.05, ** *P* < 0.01, *** *P* < 0.001.

To elucidate the mechanism underlying PCA’s effects under HG conditions, we investigated the GSK3β/Nrf2 signaling pathway and endoplasmic reticulum stress (ERS). Results demonstrated that compared to the control group, HG treatment significantly decreased the expression of phosphorylated GSK3β and Nrf2 in cells, indicating inhibition of the GSK3β/Nrf2 signaling pathway. PCA treatment significantly increased the expression of p-GSK3β and activated Nrf2 proteins (Figure 4A), suggesting that PCA activates the GSK3β/Nrf2 signaling pathway, which is suppressed by HG.To further investigate the role of the GSK3β/Nrf2 pathway in podocyte ERS, the GSK3β inhibitor TDZD-8 was employed. Western blot analysis revealed that HG treatment upregulated the expression of ERS markers C/EBP homologous protein (CHOP), glucose-regulated protein 78 (GRP78), and phosphorylated protein kinase RNA-like endoplasmic reticulum kinase (p-PERK) compared to controls. PCA intervention downregulated the expression of these proteins, suppressing podocyte ERS. However, compared to the PCA-treated group, co-treatment with TDZD-8 significantly enhanced the expression of CHOP, GRP78, and p-PERK proteins (Figure 4B). These results demonstrate that PCA inhibits endoplasmic reticulum stress in podocytes by activating the GSK3β/Nrf2 signaling pathway.

### PCA inhibits high glucose-induced podocyte inflammation and oxidative stress by suppressing endoplasmic reticulum stress *via* activation of the gsk3β/nrf2 signaling pathway

**FIGURE 5:**
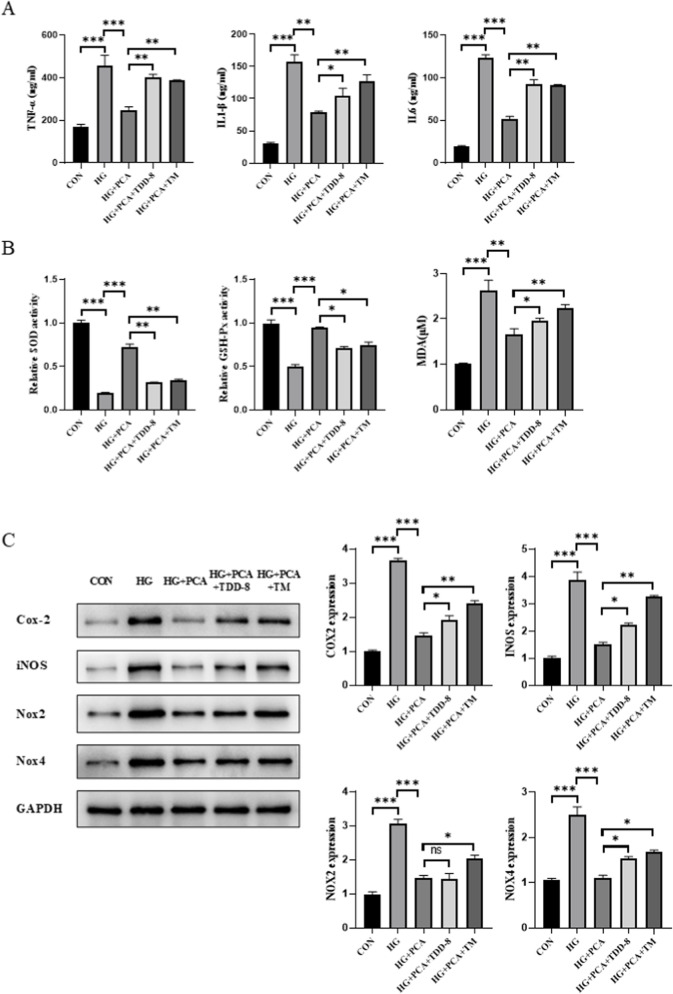
PCA inhibits high glucose-induced podocyte inflammation and oxidative stress by suppressing endoplasmic reticulum stress *via* activation of the GSK3β/Nrf2 signaling pathway. **(A)** Levels of inflammatory cytokines (TNF-α, IL-1β, and IL-6) in cells were measured using ELISA kits. **(B)** Activities of SOD and GSH-Px, as well as the content of MDA, were determined using biochemical assay kits. **(C)** Expression levels of proteins associated with inflammation and oxidative stress (Cox-2, iNOS, Nox2, and Nox4) were detected by Western blot. Data are presented as the mean ± SD, n = 3. * *P* < 0.05, ** *P* < 0.01, *** *P* < 0.001.

To further investigate the connection between the GSK3β/Nrf2 signaling pathway, ERS, inflammation, and oxidative stress, subsequent experiments were performed by adding the ERS agonist tunicamycin (TM), building upon previous findings. Consistent with the prior results, PCA intervention significantly suppressed the secretion of the inflammatory cytokines TNF-α, IL-1β, and IL-6, enhanced the activities of cellular SOD and GSH-Px, and reduced MDA content. Compared to the PCA-treated group, cells in both the TDZD-8 (a GSK-3β inhibitor) group and the TM group exhibited increased secretion of inflammatory cytokines, with the effect being more pronounced in the TM group (Figure 5A). Furthermore, the addition of TDZD-8 or TM, relative to the PCA group, reduced SOD and GSH-Px activities and increased MDA content in podocytes (Figure 5B). Concurrently, Western blot analysis (Figure 5C) demonstrated that, compared to the HG group, the expression of proteins associated with inflammation and oxidative stress was significantly decreased in the PCA group. However, compared to the PCA group, the expression of Cox-2, iNOS, Nox2, and Nox4 proteins was increased in the TDZD-8/TM groups, with this increase being more significant in the TM-treated group (Figure 5C). Taken together, these findings indicate that PCA inhibits high glucose-induced podocyte inflammation and oxidative stress by suppressing endoplasmic reticulum stress through activation of the GSK3β/Nrf2 signaling pathway.

### PCA inhibits high glucose-induced podocyte apoptosis by suppressing endoplasmic reticulum stress *via* activation of the GSK3β/Nrf2 signaling pathway

**FIGURE 6:**
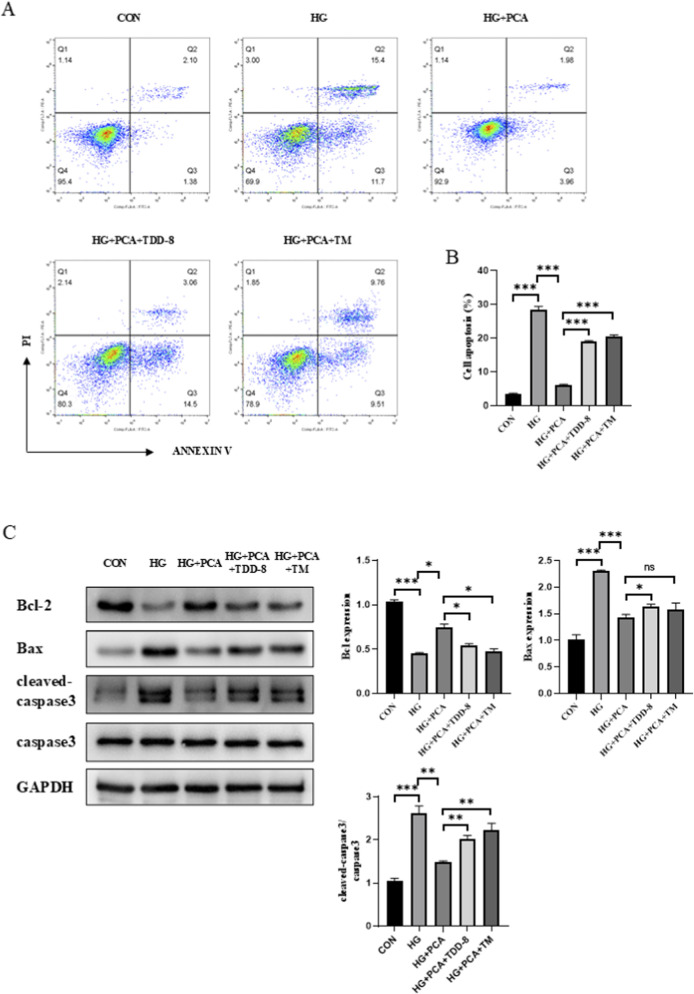
PCA inhibits high glucose-induced podocyte apoptosis by suppressing endoplasmic reticulum stress *via* activation of the GSK3β/Nrf2 signaling pathway. **(A, B)** Apoptosis was detected by flow cytometry. **(C)** The expression of apoptosis-related proteins, including Bcl2, Bax, and cleaved caspase-3, was analyzed by Western blot. Data are presented as the mean ± SD, n = 3. * *P* < 0.05, ** *P* < 0.01, *** *P* < 0.001.

Following the addition of the GSK3β inhibitor TDZD-8 and the ERS agonist TM, podocyte apoptotic levels were further assessed. Apoptosis staining results revealed that PCA downregulated the podocyte apoptosis rate. In contrast, cells in both the TDZD-8 and TM groups exhibited an increased apoptosis rate compared to the PCA group (Figure 6A). Similarly, protein detection results demonstrated that, relative to the HG group, PCA treatment increased the expression of the anti-apoptotic protein Bcl-2 while decreasing the expression of the pro-apoptotic proteins Bax and cleaved-caspase-3 in podocytes. Compared to the PCA group, cells treated with TDZD-8 or TM showed reduced Bcl-2 expression and increased expression of Bax and cleaved-caspase-3 proteins (Figure 6B). Collectively, these results demonstrate that PCA inhibits high glucose-induced podocyte apoptosis by suppressing endoplasmic reticulum stress through activation of the GSK3β/Nrf2 signaling pathway.

## Discussion

Diabetic kidney disease (DKD) represents one of the most critical complications of diabetes and a leading cause of end-stage renal disease. Podocytes, as essential components of the glomerular filtration barrier, play a pivotal role in glomerular filtration. A hyperglycemic environment induces podocyte injury and triggers apoptosis, oxidative stress, and inflammatory responses (Yang et al., 2024). Podocyte apoptosis is a key factor contributing to podocyte loss and serves as a critical cellular pathological change in the early progression of DKD (Diez-Sampedro et al., 2011). Inflammatory responses are major drivers accelerating the progression of diabetic kidney injury (Jiang et al., 2022). Inflammation persists throughout the development of DKD and is a central mechanism initiating renal damage (Rayego-Mateos et al., 2023). Inflammatory cytokines such as TNF-α, IL-1β, and IL-6 activate multiple signaling pathways, leading to insulin resistance, impaired insulin secretion, and promotion of diabetes and its complications (Xie and Du, 2011; Xue et al., 2024). Meanwhile, oxidative stress has also been implicated in the pathogenesis of DKD. Hyperglycemia promotes the formation of ROS, activating various intracellular signaling pathways that result in inflammation, apoptosis, and fibrosis (Wahab et al., 2022). The release of inflammatory cytokines is also a hallmark of oxidative stress damage and is closely associated with DKD (Domingueti et al., 2016; Liu et al., 2018). Local inflammation and oxidative stress in the kidney have been reported to induce renal cell injury and apoptosis. Moreover, metabolites released from apoptotic cells can promote inflammatory infiltration, further exacerbating kidney damage (Hu et al., 2023).

PCA’s multifaceted protection of podocytes involves suppressing NF-κB downstream signaling and inflammatory cytokine release, restoring antioxidant enzyme activities, reducing lipid peroxidation, as well as regulating the Bcl-2/Bax ratio and cleaved caspase-3 expression (Cao et al., 2022; Yang et al., 2021; Zhao et al., 2024). Previous studies have demonstrated that PCA inhibits renal inflammation and oxidative stress in DKD mice and improves renal function (Chang et al., 2021). PCA also confers protective effects against oxidative stress and restores endothelial function in streptozotocin -induced diabetic rats (Ji et al., 2021), highlighting its renal protective potential in DKD. However, the effects of PCA on podocytes in DKD remain unexplored.

GSK3β, a highly conserved serine/threonine kinase, is a key regulator in glucose metabolic pathways and plays a pivotal role in various diseases. It also serves as a biomarker reflecting DKD progression and mediates podocyte injury in DKD. GSK3β inhibitors have been shown to exert protective effects in DKD (Hou et al., 2020; Liang et al., 2024). Highly expressed in the kidney, GSK3β plays an important role in regulating self-defense mechanisms following oxidative stress by modulating the Nrf2-mediated antioxidant response (Cuadrado, 2015; Yu et al., 2020).

This study confirms for the first time that PCA improves high glucose-induced podocyte inflammation, oxidative stress, and apoptotic damage by suppressing ERS through activation of the GSK3β/Nrf2 pathway. From the perspective of the mechanism of action, our research results show: PCA can achieve podocyte protection through the “GSK3β/Nrf2-ERS axis”. First, PCA can activate the GSK3β/Nrf2 pathway. A proposed mechanism summarizing these findings is presented in the graphical abstract. This is consistent with neuroprotection research: in Parkinson’s models, PCA mediates p-GSK3β phosphorylation through PLK2, blocking its degradation of Nrf2 (Guo et al., 2019). p-GSK3β can promote nuclear translocation of Nrf2, subsequently upregulating antioxidant enzymes such as HO-1 and NQO1 (indirectly confirmed by restoration of SOD/GSH-Px activity and reduction in MDA). This mechanism is corroborated in a liver injury model: PCA enhances Nrf2 stability by inhibiting Keap1, reducing oxidative damage (Zhou et al., 2020). Second, Nrf2-mediated ERS suppression is the key bridge. PCA downregulates the pro-apoptotic protein CHOP and the ERS sensor p-PERK, while upregulating the protective chaperone protein GRP78. This is consistent with the conclusion from the TGF-β1-induced podocyte injury model: CHOP overexpression triggers caspase-12-dependent apoptosis, while GRP78 overexpression can inhibit the PERK pathway (Zhang et al., 2016). Finally, the core finding of functional validation experiments: after using the GSK3β inhibitor TDZD-8, PCA’s regulation of CHOP/GRP78/p-PERK was reversed, proving that GSK3β/Nrf2 is the upstream pathway through which PCA inhibits ERS. ERS agonist TM treatment weakens PCA’s anti-inflammatory and antioxidant effects, indicating that ERS alleviation is the prerequisite for PCA to exert its protective effect.

PCA synchronously improves inflammation, oxidative stress, and podocyte apoptosis through the GSK3β/Nrf2-ERS axis. PCA can inhibit NF-κB downstream factors and inflammatory factor release, restore SOD/GSH-Px activity and reduce lipid peroxidation. Consequently, it regulates the Bcl-2/Bax ratio and cleaved-caspase 3, ultimately reducing podocyte apoptosis. In diabetic nephropathy models, Nrf2 activation can simultaneously inhibit ROS generation and the NF-κB inflammatory pathway (Lv et al., 2023), while ERS inhibition can block the CHOP-mediated mitochondrial apoptosis pathway (Zhang et al., 2016).

Compared with similar studies, PCA has significant advantages in protecting podocytes. Compared to synthetic GSK3β inhibitors, PCA, as a natural compound, has superior safety (effective at concentrations of 1.25–5 μM in this study, with no cell toxicity), while TDZD-8, although it can protect podocytes (Liu et al., 2021), its hepatotoxicity (LD_50_ ≈ 25 mg/kg) limits clinical application. Compared to other natural phenolic acids: PCA’s renal targeting [pharmacokinetics show high kidney distribution concentration (Teng et al., 2017)] and low-dose effectiveness (significant effect at 5 μM) are superior to substances like gallic acid (often requiring >20 μM).

This study breaks through the limitations of single-pathway research, constructing for the first time in podocytes a GSK3β/Nrf2-ERS tripartite crosstalk network, proposing an integrated regulatory model of “metabolism-oxidation-protein homeostasis”, providing a new strategy for DKD treatment. Current drugs used clinically to treat diabetic nephropathy have significant limitations. SGLT2 inhibitors mainly improve glomerular hypertension and cannot directly inhibit podocyte ERS (Cai et al., 2025). RAS inhibitors although they reduce proteinuria, have difficulty blocking CHOP-dependent apoptosis (Chen et al., 2019). PCA has the potential for synergistic treatment of podocyte injury in diabetic nephropathy through multi-target action. This study confirms that PCA enhances antioxidant capacity by activating Nrf2, blocks cell apoptosis by inhibiting ERS, and reduces the secretion of inflammatory factors. In clinical treatment, the combined use of PCA and SGLT2 inhibitors may be able to synergistically protect podocytes by improving metabolic stress and alleviating ERS. Due to the lack of *in vivo* models, this study still has some limitations. Our findings are derived primarily from the MPC5 immortalized mouse podocyte line, which, while widely accepted as a model for studying podocyte biology, may not fully recapitulate the complexity of human primary podocytes or the *in vivo* microenvironment. Future studies utilizing human primary podocytes or podocyte-specific conditional knockout models would provide valuable validation of these findings and enhance their translational relevance. Simultaneously, the upstream regulatory mechanism regarding how PCA specifically activates podocyte AKT/PKC is not yet clear, and subsequent analysis needs to combine proteomics results.

In summary, this study elucidates that PCA synchronously improves oxidative damage, inflammation, and apoptosis in high glucose-exposed podocytes by reconstructing the “GSK3β/Nrf2 suppresses ERS” pathway axis. This mechanism provides a new multi-target therapeutic strategy based on natural compounds for diabetic nephropathy, expands the clinical application scenarios of PCA, deepens the understanding of the dynamic balance of the GSK3β-Nrf2-ERS network in podocyte injury, and provides a theoretical basis for targeted drug design.

## Data Availability

The raw data supporting the conclusions of this article will be made available by the authors, without undue reservation.
